# Elemental Formulas: Indications of Use in Pediatric Clinical Practice

**DOI:** 10.3390/nu17061003

**Published:** 2025-03-12

**Authors:** Sofia Zouganeli, Konstantinos Katsas, Smaragdi Fessatou

**Affiliations:** 1 Department of Nutrition and Dietetics, ATTIKON University General Hospital, 12462 Athens, Greece; szoug@yahoo.co.uk; 2 Medical School, National and Kapodistrian University of Athens, 75 Mikras Asias Street, 11527 Athens, Greece; 3 Pediatric Gastroenterology, Hepatology and Nutrition Unit, 3rd Department of Pediatrics, ATTIKON University General Hospital, 12462 Athens, Greece; sfessatou@med.uoa.gr

**Keywords:** amino acid, children, diet, elemental, formula, hydrolyzed, infants, pediatrics

## Abstract

**Background**: Elemental or amino-acid-based formulas play a crucial role in pediatric nutrition, offering a therapeutic alternative when human milk is poorly tolerated or in specific disease states. Mostly used in various cases of allergy, maldigestion, or malnutrition, elemental formulas serve as a special therapeutic regimen in clinical practice to promote growth and development. **Methods**: This narrative review briefly surveys the literature of the past decade available on PubMed, Scopus, and Google Scholar, encompassing original research, review articles, and position papers. **Results**: The indications for using elemental formulas in pediatric clinical practice extend beyond cow’s milk allergy to conditions such as eosinophilic esophagitis, intestinal failure, Crohn’s disease, hepatic failure, chronic pancreatitis, and various neurological and other malnutrition-related disorders. **Conclusions**: Elemental formulas are associated with potential health benefits for pediatric patients in certain conditions, either as a first-line feeding option or under conditional use. Despite their drawbacks, they are regarded as a therapeutic tool with numerous indications, requiring careful implementation by a multidisciplinary team of healthcare experts. Future expert guidelines, including dosage, timing, and long-term effects of elemental diets, are needed for pediatric clinical practice.

## 1. Introduction

Although human milk is universally recognized as the optimal nourishment for supporting infant growth, situations such as its unavailability or the presence of intolerance may necessitate the use of specialized formulas as an alternative [1]. Among the broad range of existing special formulas, those with hydrolyzed protein particles or free amino acids may have potential health benefits for pediatric patients experiencing maldigestion, allergy, or malnutrition-related disorders. Hydrolyzed formulas may be extensively (eHF) or partially hydrolyzed (pHF) depending on the extent of protein hydrolysis, while an elemental or amino-acid-based formula (AAF) has a specific composition based on free amino acids as the primary protein source, alongside a balanced supply of vitamins and minerals [2]. Originally described by Russell R.I. in 1975, the elemental diet generally represents an easily digestible dietary option with a low allergenic load, demonstrating efficacy across various disease states [3].

Elemental feeds are administered to pediatric patients from birth through adolescence, with their use extending across a broad range of clinical scenarios. The primary goal is to facilitate nutrient absorption, support healthy growth and development, and alleviate symptoms associated with chronic conditions linked to malnutrition or insufficient nutrient intake. Additionally, elemental diets are designed to improve gastric emptying, reduce the workload on the digestive system, and minimize gastrointestinal discomfort [4].

In this context, it is essential to explore the role of the elemental diet in pediatric care, particularly in relation to the evolving landscape of clinical practice. This narrative review synthesizes the recent literature and clinical guidelines from the past decade, providing a detailed analysis of the indications, benefits, and challenges associated with the use of elemental diets. It ultimately highlights the diverse applications of these specialized formulas in managing pediatric malnutrition and other chronic conditions.

## 2. Materials and Methods

### 2.1. Eligibility Criteria

Relevant studies, original research, review articles, position papers, and guidelines published during the last 10 years were included in our review. A study was considered eligible if any of the following criteria were met: (a) articles with infant and children populations, (b) use of elemental diet, (c) articles in the English language, and (d) articles with full-text availability. A study not considered eligible if any of the following criteria were met: (a) the retrieved article was a case report, case series, conference abstract, or expert opinion, (b) studies in teenager and adult populations, and (c) studies not published in English. If more than one study was published with the same data, only the most recent results were included.

### 2.2. Information Sources and Search Strategy

Our comprehensive literature search was conducted in PubMed, Google Scholar, and ScienceDirect databases to collect all data during the last 10 years, focused on identifying relevant studies, original research, review articles, position papers, and guidelines. The following combination of key search terms was used to gather all relevant publications: “amino acid”, “diet”, “nutrition”, “elemental”, “formula”, “hydrolyzed”, “children”, “child”, “pediatric”, “infants”, “kids”, “toddlers”, “babies”, “youth”, “neonatal”, “perinatal”, “pediatric care”, and “therapeutic”.

### 2.3. Study Selection

The study selection process was carried out by two independent authors (S.Z., K.K.) to minimize inclusion bias. After removing duplicates, the reviewers screened the titles and abstracts of all studies and determined whether each study should be included. For studies that passed this stage, the full texts were retrieved and evaluated against the inclusion criteria. Both reviewers documented their reasons for excluding studies, and in cases of disagreement, a third reviewer (S.F.) was consulted to make the final decision. Additionally, the references of all full-text articles were manually reviewed to identify any relevant articles that were not captured in the initial search results.

## 3. Discussion

### 3.1. Elemental Feeds

AAF is a low-residue, low-fat feed containing free amino acids, whereas eHF and pEF contain proteins that are either extensively or partially hydrolyzed through various techniques, such as enzymatic hydrolysis, heat pressure, and ultrafiltration [5]. Protein hydrolysis results in particles with a molecular weight (MW) ranging from less than 5–10 kDa for pHF and less than 3 kDa for eHF [6]. However, a MW of 1.2 kDa for eHF has been proposed as the cutoff for allergenicity to eliminate all potential residual allergens [7]. AAF excludes lactose, using monosaccharides and maltodextrins as the main carbohydrate sources, and incorporates medium-chain triglycerides (MCTs) as the main lipid source, along with essential fatty acids [8].

**Figure 1:**
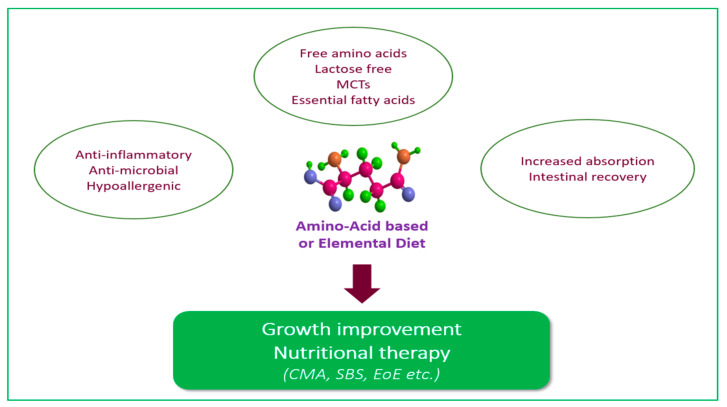
Elemental diet characteristics and actions. Abbreviations. Medium-chain triglycerides (MCTs); Cow’s milk allergy (CMA); Short bowel syndrome (SBS); Eosinophilic Esophagitis (EoE).

One of the primary benefits of elemental formulas is their low allergenicity. Since they consist of free amino acids, they are less likely to trigger allergic reactions compared to intact protein formulas, being widely preferred in cases of food intolerance and protein allergies. They have also proven effective in cases of maldigestion, as they contain easily digestible MCTs and are low residue, alleviating symptoms such as gastrointestinal distress, bloating, diarrhea, and other issues related to digestive discomfort [4]. In addition, free amino acids may have anti-inflammatory properties and act as gut modulators to promote mucosal healing [4,9] (Figure 1), as seen in cases such as eosinophilic esophagitis (EoE) and Crohn’s disease (CD) [10,11]. Elemental formulas have been employed as a first-line treatment for certain conditions, as well as a therapeutic option when other treatments have proven ineffective (Table 1). The primary objective, in any case, is to improve malnutrition indices and foster growth and development. 

**Figure 2:**
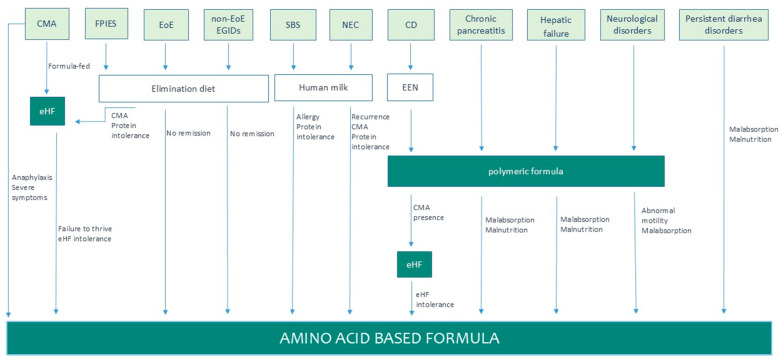
Schematic decision process for appropriate formula selection. Abbreviations: Cow’s milk allergy (CMA); Food-protein induced enterocolitis syndrome (FPIES); Extensively hydrolyzed formula (eHF); Eosinophilic Esophagitis (EoE); non-EoE EGIDs (non-EoE Gastrointestinal disorders); Crohn’s disease (CD); Necrotizing enterocolitis (NEC); Short Bowel Syndrome (SBS); Exclusive enteral nutrition (EEN).

In comparison to elemental feeds, polymeric and hydrolyzed formulas contain intact protein or small peptides of different molecular weights, respectively, showing effectiveness across a broad range of conditions requiring nutritional support. For instance, iso-osmolar polymeric formulas have been recommended for the initiation of feeding after PEG gastrostomy insertion [12], in undernourished children with cystic fibrosis [13], in various neurological disorders [14] and in exclusive enteral nutrition (EEN) for pediatric patients with CD [11], or for certain cases of chronic pancreatitis [15] and hepatic failure [16] (Figure 2). EHF has been suggested as a hypoallergenic option, mainly in children with cow’s milk allergy (CMA), food protein-induced enterocolitis syndrome (FPIES), and in other cases such as CD (Figure 2).

### 3.2. Necrotizing Enterocolitis (NEC)

NEC, initially described by Mizrahi et al. in 1965, is characterized by mild to severe intestinal inflammation and is strongly associated with low gestational age (<35 weeks) [17]. Factors such as gut immaturity, immunological processes associated with prematurity, and the observed low diversity of gut microbiota in preterm infants may compromise intestinal permeability and contribute to inflammation [18].

In infants recovering from NEC, human milk is the first choice due to its benefits in enhancing digestibility, immunity, and intestinal adaptation [19]. However, in the absence of human milk or in cases of intolerance, elemental feeds containing easily absorbed MCTs may be used to promote growth despite the lack of robust evidence [20]. Additionally, in instances where symptoms of intolerance persist or there is a recurrence of NEC episodes, which may also suggest a diagnostic overlap between CMA and NEC, preterm formula-fed infants may also benefit from consuming an elemental feed [21].

### 3.3. Short Bowel Syndrome

Pediatric intestinal failure (IF) is defined as “the requirement for parenteral nutrition (PN) for more than 60 days due to intestinal disease, dysfunction, or resection” [22]. Short bowel syndrome (SBS), the primary cause of IF, is defined as “the need for PN for more than 60 days following intestinal resection or when bowel length is less than 25% of expected” [22].

While PN serves as the primary dietary intervention to support recovery and growth, enteral nutrition is gradually introduced to promote intestinal adaptation, with the ultimate goal of PN weaning [23]. Human milk is widely recognized as the preferred and most beneficial nutritional option for infants with intestinal failure due to its numerous health benefits, including optimal growth and immune support [24,25].

Infants with increased intestinal permeability, a condition often associated with SBS, may be more susceptible to the development of food allergies due to the impaired barrier function of the gut [24]. This heightened vulnerability can lead to an increased risk of allergic reactions to proteins and other allergens present in food [26]. In such cases, elemental formulas may be among the selected options with significant benefits.

### 3.4. Cow’s Milk Allergy (CMA) and Food Protein Induced Enterocolitis Syndrome (FPIES)

Cow’s milk proteins can trigger IgE, non-IgE, or mixed immunological reactions upon consumption [27], leading to a range of symptoms. These symptoms include skin reactions, such as an itchy rash or swelling of the lips, face, and around the eyes, as well as atopic eczema, while digestive issues, such as vomiting, gastroesophageal reflux disease (GERD), colic, diarrhea, constipation, abdominal pain, anorexia, and dysphagia, along with respiratory reactions like a runny nose and wheezing, may occur [28]. In FPIES, a non-IgE cell-mediated food allergy, such symptoms depend on the type and quantity of the ingested food allergen, as well as the child’s age and phenotype [29]. Whether acute or chronic, FPIES necessitates direct nutritional intervention. It is essential to identify the triggering food and implement a supervised and personalized elimination diet to alleviate symptoms and manage potential hypoalbuminemia and growth delay. In preterm infants, avoidance of cow’s milk protein is necessary since it is the most common triggering factor [21].

In cases where intolerance is present, formula-fed infants with CMA are typically offered eHF [30]. This choice is based on the understanding that small peptides with a MW of less than 1.2–1.5 kDa are more hypoallergenic compared to intact protein and peptides of MW less than 5 kDa, which are found in partially pHF [28]. In breastfed infants, although CMA is rare, a cow’s milk elimination diet is suggested to mothers for 2 to 4 weeks while breastfeeding, with continuous diet monitoring to prevent or manage nutritional deficiencies and proper reintroduction based on the infant’s symptoms [30]. However, human milk itself may provide the necessary immunological factors to lower severe symptoms of CMA-related atopic eczema/dermatitis syndrome, as breastfeeding has been associated with a significant increase in IL-10, a regulator of immunological response [31].

AAF remains the sole antiallergenic option with established efficacy for infants and children with CMA who do not tolerate eHF after a minimum of two weeks of consumption or exhibit severe clinical symptoms, including anaphylaxis, according to the EAACI Food Allergy and Anaphylaxis Guidelines [32]. Most recently, guidelines for CMA by the World Allergy Organization (WAO DRACMA Update Guidelines) also suggest that an AAF should be the second option for both IgE or non-IgE mediated CMA patients after an eHF unless severe symptoms exist [33]. The presence of nutritional deficiencies and the growth rate have been under consideration when comparing different formulas, with some evidence favoring the elemental feeds as to improved growth in children with CMA [34]. Regarding the nutritional composition of AAF, it is suggested that a specific ratio of 3–4.5 g of protein equivalent per 100 kcal is essential for promoting protein anabolism and minimizing the risk of increased nitrogen excretion [27].

### 3.5. Eosinophilic Esophagitis (EoE)—Eosinophilic Gastrointestinal Disorders (EGIDs)

EoE is an immune-mediated disease characterized by the infiltration of eosinophils into the esophagus, considered significant when the eosinophil count exceeds 15 eosinophils (eos) per high-power field (HPF) in biopsies from the proximal and/or distal esophagus [35]. This condition, commonly observed in infants and children, manifests with symptoms such as dysphagia, slow eating, choking, and abdominal pain [36].

In addition to pharmacological treatment, three dietary approaches have been proposed for managing EoE: the elemental diet, the empirical elimination diet, and the allergy test-directed elimination diet [10]. The allergy test-directed elimination diet, which involves removing specific foods based on positive blood or skin allergy tests, is considered the least effective dietary approach, with a histological remission rate of 39.5% [37]. In comparison, the empirical elimination diet shows higher efficacy, with a histological remission rate of 64% after the removal of six foods and 44–55% after eliminating one to four foods [37]. The elemental diet, first found effective in 1995 by Kelly et al. [38], has subsequently been shown in meta-analyses to achieve histological remission in 96% of pediatric EoE patients after four to eight weeks of implementation [37,39]. Unlike elimination diets, the elemental diet is devoid of food allergens that may trigger EoE inflammation [40].

Despite its efficacy, the elemental diet has several drawbacks, including its expense, poor palatability, and negative impact on children’s quality of life. As a result, ESPGHAN recommends considering the elemental diet only after other treatments have failed, particularly for formula or tube-fed infants and children or those who have not achieved remission with other dietary therapies [10].

Recent joint guidelines from ESPGHAN and NASPGHAN for patients with non-EoE EGIDs, such as eosinophilic gastritis, gastroenteritis, and colitis, recommend conditional empiric food allergy elimination diets as the preferred approach for selected patients [41]. Elemental feeds have been shown to be effective in 76% of children with eosinophilic gastroenteritis, according to a systematic review of 30 studies, but results were reported in terms of clinical and not histological remission [42]. Other pediatric patients with eosinophilic gastritis, gastroenteritis, and colitis were also offered an elemental diet, among other proposed dietary interventions resulting in clinical improvement [43]. In both cases, findings were associated with the effectiveness of the elemental diet without leading to a routine dietary approach due to the lack of strong evidence [41].

### 3.6. Inflammatory Bowel Disease

Exclusive enteral nutrition (EEN) administered for a duration of 6 to 8 weeks has been demonstrated to be an effective therapeutic approach for inducing remission in pediatric patients with active luminal Crohn’s disease (CD), with evidence suggesting it may be more efficacious than corticosteroids in this population [44].

Various types of formulas, either polymeric, elemental, or semi-elemental, have been used during EEN therapy with effectiveness in inducing remission, promoting mucus healing, and supporting growth [11]. According to a Cochrane systematic review [45] and the most recent ECCO-ESPGHAN guideline update [46], there is no statistically significant difference in remission or relapse rates when comparing different formulas based on their protein content. This includes comparisons between elemental vs. polymeric formulas, as well as elemental vs. semi-elemental formulas. Despite the extended use of elemental diet in CD patients, there is insufficient evidence to support its routine use unless comorbid conditions, such as CMA, are present [11].

Interesting results concerning the superiority of elemental versus polymeric formulas have been recently reported as to the remission of colitis in mice through the reshaping of gut microbiota and the concomitant inhibition of mucus layer disruption, invasion, and degradation [9]. This study highlights that microbiota serve as mediators of the effectiveness of the elemental diet in relation to the progression of colitis and the development of inflammation, thereby contributing to future therapeutic nutritional approaches.

### 3.7. Neurological Disorders

Children with neurological disorders often experience gastrointestinal symptoms, including delayed gastric emptying, malabsorption, gastroesophageal reflux disease (GERD), diarrhea, or constipation [47]. These issues can lead to feeding difficulties and nutritional deficiencies [48]. In patients where abnormal motility is present, elemental formulas are included in the recommended regimes.

Although no special formula has been suggested to support the nutritional status of patients with neuromuscular disorders like spinal muscular atrophy (SMA) or Duchenne Muscular Dystrophy and other [49], in some cases of children with spinal muscular atrophy type I, elemental formulas have been associated with a positive impact on reflux symptoms, motility, abdominal distension, gastric emptying time, and constipation, especially when combined with low-fat content [50,51]. To mention, patients with neurological disorders with gastrointestinal manifestations and food intolerance often need long-term enteral nutritional support even at home [52], with the enteral feeding regime started in-hospital and continued at home under the guidance of the healthcare team.

Conflicting results have emerged regarding the ideal type of feed for pediatric patients with neurological conditions, primarily due to factors such as the small number of patients included in studies, differences in study design, the complex nature of neurological disorders, and the variability in diagnoses [8]. As a result, it is increasingly recognized that a more individualized approach to nutrition is necessary, one that takes into account the specific needs, underlying conditions, and responses to treatment of each patient. Such a personalized strategy would allow for optimal management of both nutritional requirements and the neurological condition itself, ultimately improving patient outcomes.

### 3.8. Other Indications

#### 3.8.1. Hepatic Failure

Chronic liver disease often coincides with a heightened risk of malnutrition. Children with cholestatic or end-stage liver diseases commonly experience fat malabsorption, resulting in steatorrhea, increased protein catabolism, and deficiencies in fat-soluble vitamins. Hence, immediate provision of energy, fat, and protein is crucial to support growth and enhance health outcomes. According to the 2019 position paper by ESPGHAN and NASPGHAN, the recommended energy intake for children should be 130% of age-related requirements, protein intake should range between 130–150% of age-related requirements, carbohydrates should constitute 40–60% of energy, and fat should account for 30–40% of energy [16]. MCT supplements are strongly advised in cholestasis, but their proportion should not exceed 80% of total fat to prevent essential fatty acid deficiency, with an ideal MCTs/long-chain triglycerides (LCTs) ratio of 30%/70% of total fat and under dietary guidance to limit the risk of steatorrhea [16]. MCT sources include oil supplements and MCT-enriched formulas. Many formulas containing MCTs are elemental or hydrolyzed, making them viable options for children with chronic liver disease, particularly those requiring nasogastric or nasojejunal feeding, despite the lack of robust evidence.

#### 3.8.2. Chronic Pancreatitis (CP)

The potential presence of pancreatic lipase insufficiency in children with chronic pancreatic failure can result in fat maldigestion and subsequent malnutrition [52]. While a regular diet is generally recommended for children with chronic pancreatitis, MCT formulas may be conditionally recommended for those needing to enhance their nutritional status [52]. This recommendation stems from the fact that MCTs are more easily absorbed without requiring the same amount of pancreatic lipase as LCTs [53]. In addition to incorporating MCT oil into the regimen, MCTs can be obtained through elemental or semi-elemental formulas, particularly suitable for infants. In such cases, the use of elemental formula may prove effective in improving weight gain by facilitating better fat digestion.

#### 3.8.3. Persistent Diarrhea Disorders

For pediatric populations with enduring persistent diarrhea and malnutrition stemming from infectious and gastrointestinal diseases, the utilization of elemental formulas has been linked with enhanced weight gain, improved intestinal absorptive capacity, and intestinal recovery and may be linked to improved stool frequency and consistency, and reduction of the necessity for parenteral nutrition [54]. These results may be attributed to the low antigen load of elemental formulas and the subsequent increase in nutrient absorption and calorie intake [54].

## 4. Disadvantages—Side Effects

While the elemental diet has shown significant benefits in improving growth and enhancing the quality of life in children (Table 1), it comes with several disadvantages.

Firstly, elemental feeds tend to be more expensive due to the intricate processes involved in protein hydrolysis, as reported in studies examining the cost-effectiveness of specialized formulas [55,56].

The distinct taste of AAF is often poorly tolerated by children and adolescents, leading to low compliance, while there is frequently the need for feeding tube placement [10,57].

Additionally, the elemental diet may interfere with the developmental process of eating, especially in younger children, and delay the introduction of foods in the first year of life, potentially leading to future IgE-mediated food allergies [35].

Moreover, AAF may not be well tolerated due to its high osmolality [58], which can lead to symptoms such as diarrhea, vomiting, or dumping syndrome.

The use of elemental formulas may lead to hypophosphatemia and related complications, such as fractures or rickets, stemming from impaired phosphorus absorption [59]. This necessitates careful monitoring, potential phosphorus supplementation, or consideration of alternative formulas to mitigate symptoms.

## 5. Limitations

This narrative review has several limitations. First, a non-systematic literature review is more prone to bias and subjectivity, as it lacks a standardized methodology and structured search strategy. Additionally, it does not always critically assess study quality, making the findings less reliable and harder to reproduce. This can result in incomplete coverage and overgeneralized conclusions. However, this study aimed to provide a broad overview of the indications for elemental formula use in pediatric clinical practice rather than to evaluate specific interventions or outcomes. Secondly, while an effort was made to reference various disease states, there is a risk that some conditions in which elemental diets may be effective were not included. Another limitation is the lack of in-depth analysis of the long-term effects of elemental formulas and their impact on pediatric growth, neurodevelopment, and metabolic health, while no clinical monitoring strategies or risk management guidelines are provided. As an additional limitation, our review fails to provide appropriate dosage guidelines and nutritional monitoring indicators, omitting essential details on assessing growth, micronutrient status, and bone health in long-term use. Future expert guidelines for preventing hypophosphatemia and ensuring adequate nutrient supplementation are needed in pediatric clinical practice. Additionally, there is still a lack of high-quality, evidence-based clinical trials and expert guidelines to support pediatric clinical practice regarding the optimal timing and method of elemental diet implementation (i.e., the literature on the use of elemental formulas for hepatic failure seems to be very scarce). As a result, our findings are restricted to indications rather than strong recommendations or suggested guidelines. Therefore, the presented indications of elemental formula utilization aim to contribute to clinicians’ decisions for better patient therapeutic management.

## 6. Conclusions

The elemental diet has demonstrated efficacy in managing a range of disorders characterized by nutrient malabsorption, disrupted gut motility, food intolerances, or allergic reactions. Whether the need for an elemental formula arises directly from a clinical condition or as an indirect therapeutic strategy, there remain numerous unresolved questions that warrant further investigation. Key areas for future research include determining the optimal timing and duration for implementing the elemental diet, ensuring adequate nutrient sufficiency during prolonged use, and evaluating the long-term impact of the diet on growth and development. Despite these gaps in knowledge, the elemental diet continues to play a crucial role in the nutritional management of complex clinical cases, particularly when guided by an expert multidisciplinary healthcare team and serves as a valuable therapeutic tool in pediatric clinical practice.

## Figures and Tables

**Table 1:** Indications of elemental formula use in pediatric clinical practice.

First-Line Indications	Second-Line Indications
✔ Symptoms of anaphylaxis (CMA)	✔ Chronic liver disease needing MCTs through elemental or hydrolyzed formula
✔ Severe clinical symptoms and failure to thrive (CMA, FPIES)	✔ Chronic pancreatitis: support nutritional status and pain reduction through MCT-elemental or hydrolyzed formula
✔ CMA not tolerating eHF	✔ Recurrent episodes of NEC
✔ CD with presence of CMA	✔ Abnormal motility in pediatric neurological disorders
✔ EoE and other non-EoE disorders after failure of properly performed medical treatment and/or elimination diet	✔ Persistent diarrhea disorders
✔ SBS with the presence of allergy or protein intolerance	

Abbreviations. Cow’s milk allergy (CMA); Food-protein induced enterocolitis syndrome (FPIES); Extensively hydrolyzed formula (eHF); Eosinophilic Esophagitis (EoE); Crohn’s disease (CD); Necrotizing enterocolitis (NEC); Short Bowel Syndrome (SBS); Medium-chain triglycerides (MCTs).

## Abbreviations

The following abbreviations are used in this manuscript:

AAF	Amino acid-based formulas
CMA	Cow’s milk allergy
CD	Crohn’s disease
eHF	Extensively hydrolyzed formula
EGIDs	Eosinophilic Gastrointestinal Disorders
EoE	Eosinophilic Esophagitis
GERD	Gastroesophageal reflux disease
FPIES	Food-protein-induced enterocolitis syndrome
LCTs	Long-chain triglycerides
MW	Molecular weight
MCTs	Medium-chain triglycerides
NEC	Necrotizing enterocolitis
PN	Parenteral nutrition
IF	Pediatric intestinal failure
SBS	Short bowel syndrome
WAO	World Allergy Organization
